# Chronic treatment with prazosin or duloxetine lessens concurrent anxiety-like behavior and alcohol intake: evidence of disrupted noradrenergic signaling in anxiety-related alcohol use

**DOI:** 10.1002/brb3.230

**Published:** 2014-04-14

**Authors:** Mary J Skelly, Jeff L Weiner

**Affiliations:** Department of Physiology and Pharmacology, Wake Forest School of Medicine, Medical Center BoulevardWinston-Salem, North Carolina, 27157

**Keywords:** Duloxetine, osmotic minipump, plus maze, prazosin, propranolol, stress

## Abstract

**Background:**

Alcohol use disorders have been linked to increased anxiety, and enhanced central noradrenergic signaling may partly explain this relationship. Pharmacological interventions believed to reduce the excitatory effects of norepinephrine have proven effective in attenuating ethanol intake in alcoholics as well as in rodent models of ethanol dependence. However, most preclinical investigations into the effectiveness of these drugs in decreasing ethanol intake have been limited to acute observations, and none have concurrently assessed their anxiolytic effects. The purpose of these studies was to examine the long-term effectiveness of pharmacological interventions presumed to decrease norepinephrine signaling on concomitant ethanol self-administration and anxiety-like behavior in adult rats with relatively high levels of antecedent anxiety-like behavior.

**Methods:**

Adult male Long-Evans rats self-administered ethanol on an intermittent access schedule for eight to ten weeks prior to being implanted with osmotic minipumps containing either an a1-adrenoreceptor antagonist (prazosin, 1.5 mg/kg/day), a *β*1/2-adrenoreceptor antagonist (propranolol, 2.5 mg/kg/day), a serotonin/norepinephrine reuptake inhibitor (duloxetine, 1.5 mg/kg/day) or vehicle (10% dimethyl sulfoxide). These drugs were continuously delivered across four weeks, during which animals continued to have intermittent access to ethanol. Anxiety-like behavior was assessed on the elevated plus maze before treatment and again near the end of the drug delivery period.

**Results:**

Our results indicate that chronic treatment with a low dose of prazosin or duloxetine significantly decreases ethanol self-administration (*P* < 0.05). Furthermore, this decrease in drinking is accompanied by significant reductions in the expression of anxiety-like behavior (*P* < 0.05).

**Conclusions:**

These findings suggest that chronic treatment with putative inhibitors of central noradrenergic signaling may attenuate ethanol intake via a reduction in anxiety-like behavior.

## Introduction

The strength of the relationship between alcohol use and anxiety disorders has been extensively documented, and these commonly comorbid conditions are recognized as being among the most prevalent psychiatric diseases afflicting the United States population (Grant et al. 2004). Alcohol is a potent anxiolytic, and anxiety promotes drinking in both humans and animals (Khantzian 1985; Spanagel et al. 1995; Sayette 1999; Willinger et al. 2002). Paradoxically, ethanol withdrawal results in increased anxiety, and this anxiety is potentiated by cyclic intoxication and withdrawal (Becker 2012); thus, withdrawal-related anxiogenesis may promote further drinking and contribute to the development of alcohol dependence (Koob 2013). In support of this, animal studies suggest that the anxiety resulting from extended alcohol exposure persists despite cessation of intake (Valdez et al. 2002; Santucci et al. 2008), and long-abstinent alcoholics complain of enduringly increased anxiety (Willinger et al. 2002; Adinoff et al. 2005). In fact, the severity of anxiety during sobriety can be prognostic of relapse to alcohol abuse (Willinger et al. 2002; Sloan et al. 2003), and alcoholics cite anxiety as a major impetus for reversion to drinking (Sinha et al. 2011). As such, identifying pharmacological interventions which target the neurobiological substrates common to anxiety and alcohol abuse may prove crucial to successfully treating alcohol dependence.

Previous investigations aimed at identifying the neuroadaptations pertinent to this comorbidity have revealed a potential role for central noradrenergic dysregulation in both anxiety and alcohol use disorders. For example, over activity of the noradrenergic system contributes to anxiety disorders (Yamamoto et al. 2014), and cyclic alcohol intake and withdrawal further potentiates noradrenergic signaling (Becker 2012). Both anxiety and alcohol intake influence activity of the locus coeruleus (LC), a brain region crucial to coordinating the behavioral stress response. The LC's profuse noradrenergic afferents fire in response to stress (Pavcovich et al. 1990), and anxiety results in part from chronic stress-induced potentiation of LC output (Ressler and Nemeroff 2001). Ethanol likewise increases LC firing (Aston-Jones et al. 1982), and evidence from both clinical and preclinical investigations suggests that noradrenergic signaling is persistently elevated subsequent to chronic ethanol (Hawley et al. 1994; Patkar et al. 2003, 2004; Rasmussen et al. 2006).

Consistent with these data, pharmacological interventions presumed to decrease noradrenergic signaling have proven effective in attenuating alcohol self-administration in both human alcoholics and rodent models of ethanol dependence. For example, the *α*-1 adrenergic receptor antagonist prazosin decreases drinking and stress-induced alcohol craving in alcoholics (Simpson et al. 2009; Fox et al. 2012) and reduces ethanol self-administration in rodent models of ethanol dependence (Walker et al. 2008; Rasmussen et al. 2009; Verplaetse et al. 2012). Similarly, *β*-1/2 adrenoreceptor antagonists have been used clinically to treat anxiety associated with alcohol withdrawal (Sellers et al. 1977; Bailly et al. 1992), and also decrease operant responding for ethanol in ethanol-dependent rats (Gilpin and Koob 2010). Serotonin and norepinephrine reuptake inhibitors (SNRIs) have also shown promise in reducing anxiety-related alcohol intake, reportedly lessening alcohol craving and anxiety following withdrawal in clinical populations (Liappas et al. 2005; Kim et al. 2006; Petrakis et al. 2012) and likewise attenuating ethanol self-administration and the symptoms of acute withdrawal in rodents (Saglam et al. 2004; Ji et al. 2008; Simon O'Brien et al. 2011). Finally, recent findings from our lab suggest that prazosin, the *β*-1/2 adrenoreceptor antagonist propranolol, and the SNRI duloxetine acutely decrease ethanol self-administration in adult rats expressing high levels of anxiety-like behavior (Skelly et al. 2012). Thus, the effectiveness of various noradrenergic pharmacological agents in treating alcohol dependence and related anxiety appears promising.

To date, preclinical investigations into the effectiveness of noradrenergic pharmacotherapeutics in attenuating drinking-related behaviors have largely been limited to acute observations, and none have concurrently examined their anxiolytic effects. Although existing studies have greatly expanded our understanding of NE deregulation in anxiety-related alcohol dependence, a robust animal model of these disorders should replicate the decreased anxiety and ethanol self-administration observed in human alcoholics following chronic treatment with agents that decrease NE signaling. Furthermore, concomitant comparisons of drug efficacy on anxiety and ethanol intake in the same animals provides the opportunity to strengthen or disprove the notion that ethanol's anxiolytic effects contribute to ethanol dependence. Two recently published reports have found that chronically administered prazosin does in fact decrease rodent ethanol self-administration (Froehlich et al. 2013a,b); these important studies are the first to mimic the observed effects of this drug among clinical populations (Simpson et al. 2009; Fox et al. 2012). However, further research is needed to determine the long-term effectiveness of noradrenergic agents on concurrent ethanol intake and anxiety-like behavior in established rodent models, as identifying the alterations that occur following continual treatment with these drugs may elucidate the underlying disruptions to the noradrenergic system ensuing from excessive drinking. The experiments presented herein replicate and extend the recent findings by Froehlich and colleagues by examining the effects of chronically administered prazosin, propranolol, and duloxetine on both ethanol self-administration and anxiety-like behavior in adult male Long-Evans rats known to exhibit an anxiogenic phenotype (Chappell et al. 2013). We report that not only do prazosin and duloxetine significantly decrease ethanol intake, but that this decrease in drinking is accompanied by significant reductions in the expression of anxiety-like behavior. These findings suggest that decreased drinking following long-term treatment with putative inhibitors of central noradrenergic signaling may result from treatment-induced attenuation of anxiety-like behavior.

## Materials and Methods

All experiments were conducted in accordance with the guidelines set forth by the National Institutes of Health Guide for the Care and Use of Laboratory Animals, and were approved by the Wake Forest University Institutional Animal Care and Use Committee. Thirty-six adult male Long-Evans rats (Harlan, Indianapolis, IN) were obtained weighing approximately 300 g and were individually housed in standard polypropylene shoebox cages. Subjects were maintained on a 12-hour light/dark schedule (lights on at 7 am) with continuous access to rodent chow and water.

### Behavioral procedures

**Figure 1:**
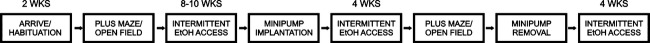
Experimental timeline. Adult male Long-Evans rats were singly housed upon arrival and allowed to acclimate to the environment for 2 weeks. Following this, baseline anxiety-like behavior was assessed using the elevated plus maze and open field tests. Animals were then given intermittent access to ethanol (20% v/v) and water 3 days a week (MWF) for 24 h; this homecage drinking continued for 8–10 weeks. Animals were weighed prior to ethanol access on drinking days. Following this intermittent access period, all animals underwent a surgical procedure during which osmotic minipumps were implanted containing either vehicle (10% DMSO in sterile saline), propranolol (2.5 mg/kg/day), prazosin (1.5 mg/kg/day), or duloxetine (1.5 mg/kg/day). Animals were then allowed to continue intermittent access homecage drinking for four additional weeks. During the last week, animals were again exposed to the elevated plus maze and open field on nondrinking days. Animals underwent a second surgery to remove the osmotic minipumps, and were again exposed to the drinking procedure for 4 weeks prior to being sacrificed.

Following 2 weeks of acclimation to the housing environment, baseline anxiety-like behavior was assessed using elevated plus mazes (Med Associates, St. Albans, VT) (Fig. 1). The mazes were elevated 72.4 cm from floor level, with runways measuring 10.2 cm wide by 50.8 cm long. Open runways had 1.3 cm high lips and closed runways were enclosed in 40.6 cm high black polypropylene walls. Exits and entries from each runway were detected via infrared sensors attached to the opening of each arm of the maze. Data were obtained and recorded via personal computer interfaced with control units and MED-PC programming (Med Associates). Animals were placed at the junction of the four arms at the beginning of the session, and activity was measured for 5 min. Anxiety-like behavior was assessed by measuring the total time spent on the open arms of the maze as well as the total percentage of entries into the open arms. General locomotor activity was assessed by measuring the total number of closed arm entries.

On a separate day, anxiety-like behavior and general locomotion were measured using an open field test, conducted in standard activity chambers (model-RXYZCM, Digiscan animal activity monitors, Omnitech, Columbus, OH) (Fig. 1). At the start of the test, animals were placed in the center of acrylic plastic chambers (42 * 42 * 30 cm) equipped with eight photobeam arrays of infrared photodectors located at regular intervals along each wall of the chamber (2.5 cm above the floor). Exploratory activity in this environment was measured for one hour, and data were stored in five minute time bins. Locomotor behavior was assessed by measuring the total distance traveled during this time, while anxiety-like behavior was assessed as the percentage of time spent in the center of the chamber relative to the perimeter.

Following this, ethanol consumption was measured for 8–10 weeks (24–30 ethanol access sessions) using an intermittent access two-bottle choice procedure (20% ethanol [v/v]; water) (Fig. 1). Animals were given access to ethanol and water for 24 h every Monday, Wednesday, and Friday, with only water available on the remaining days of the week. On ethanol drinking days, fluid intake was assessed following the first 30 min of access and again at 24 h. At the end of 8 weeks, animals were divided into matched groups according to their daily ethanol intake and baseline anxiety-like behavior on the elevated plus maze. Over the subsequent 2 weeks, all animals underwent surgery to implant osmotic minipumps which delivered either vehicle or treatment compounds (see “surgical procedures”). Intermittent ethanol access continued for 4 weeks following minipump implantation, as described above. At the end of the fourth week of drug delivery, anxiety-like behavior and general locomotion were again assessed in the elevated plus maze and open field prior to minipump removal. Following treatment cessation, intermittent ethanol access continued for at least four additional weeks; animals were then sacrificed via sodium pentobarbital overdose.

### Drug dosing

Drug doses were calculated based on the estimated mean weight of animals in each group halfway through the drug delivery period (taking the mean weight at baseline and adding projected weight gain across 2 weeks). Propranolol (1.275 mg/day, or ∼2.5 mg/kg/day), prazosin (0.78 mg/day, or ∼1.5 mg/kg/day), and duloxetine (0.75 mg/day, or ∼1.5 mg/kg/day) were obtained from Tocris Bioscience (Minneapolis, MN). These doses were observed to be effective in reducing ethanol intake among stressed rats when administered via acute i.p. injection (Skelly et al. 2012); thus, the rough equivalent of an acutely effective bolus injection was infused across a 24-h period. All drugs were dissolved in dimethyl sulfoxide (DMSO) prior to being diluted down to their final concentrations in sterile saline (0.9% NaCl). Vehicle solution contained 10% DMSO in sterile saline. Drugs were loaded into Alzet osmotic minipumps (Model 2ML4) in strict accordance with manufacturer specifications.

### Surgical procedures

Animals were anesthetized with an i.p administered cocktail of ketamine (100 mg/kg) and xylazine (10 mg/kg). Standard aseptic surgical procedures were used to implant osmotic minipumps subcutaneously. Briefly, the area between the shoulder blades was shaved and cleaned. A small incision was made in the skin between the scapulae, and hemostats were used to create a pocket in the subcutaneous tissue into which the minipump was inserted. The incision was sutured shut and animals were administered ketoprofen (3 mg/kg, s.c.) to attenuate postsurgical discomfort. Minipumps remained in place for 4 weeks, at which point the animals were again anesthetized following the above procedure and pumps were removed. Any remaining drug solution was extracted from each pump following surgery, to ensure proper dispersal of solution across the 4-week treatment period.

### Statistical analysis

During sessions where ethanol was available, each animal's daily ethanol consumption (24 h g/kg), binge-like intake (g/kg in the first 30 min of daily access), and 24 h preference for ethanol relative to water were measured. Daily measures were averaged across each week of drinking prior to analysis, and the final week of drinking prior to drug or vehicle treatment served as a baseline measure for subsequent analyses. Between group changes in ethanol intake and preference across all groups were assessed first via two-way repeated measures analysis of variance (ANOVA), and then via separate two-way repeated measures ANOVAs comparing animals in each treatment group to vehicle-treated conspecifics. Within-treatment changes in intake measures were assessed via one-way repeated measures ANOVAs comparing baseline drinking to each week of drug treatment. To assess whether changes in intake persisted following cessation of treatment, one-way repeated measures ANOVAs were run comparing intake during the final week of drug treatment to each post-treatment week. Follow-up Newman–Keuls post hoc analyses were run when appropriate. Anxiety-like behavior on the elevated plus maze was analyzed via one-way ANOVAs and Newman–Keuls post hoc analyses comparing all treatment groups at baseline and again following 4 weeks of treatment, and activity in the open field test was analyzed using two-way repeated measures ANOVAs comparing treatment groups across time at baseline and again during the final week of drug treatment. Finally, a Pearson correlation was run analyzing the relationship between time in the open arms of the elevated plus maze after drug treatment and drinking during week four of drug treatment relative to baseline. The significance level for all statistical analyses was set at *P* < 0.05.

## Results

### Prazosin and duloxetine decrease anxiety-like behavior

**Figure 2:**
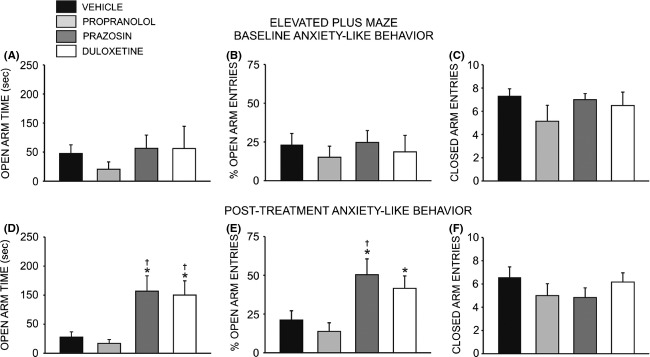
Chronic treatment with prazosin or duloxetine decreases anxiety-like behavior on the elevated plus maze. Top bar graphs illustrate that neither anxiety-like behavior, measured as time spent on the open arms of the maze (A) and total number of open arm entries (B), nor general locomotor activity (C) (assessed as number of closed arm entries) differed significantly between groups of adult male Long-Evans rats at baseline (one-way ANOVAs, *P* > 0.05). Bottom bar graphs illustrate that 4 week treatment with prazosin (*n* = 6, 1.5 mg/kg/day) or duloxetine (*n* = 6, 1.5 mg/kg/day) via osmotic minipump selectively increased time spent on the open arms of the maze (D) and total number of open arm entries (E), reflecting a decrease in anxiety-like behavior while having no effect on general locomotor activity (F) (*, significant difference relative to propranolol-treated animals (*n* = 7, 2.5 mg/kg/day), one-way ANOVAs and Newman–Keuls post hoc tests, *P* < 0.05; †, significant difference relative to vehicle-treated animals (*n* = 7, 10% DMSO), one-way ANOVAs and Newman–Keuls post hoc tests, *P* < 0.05).

All animals were exposed to the elevated plus maze and open field test, and then allowed to self-administer ethanol for 8–10 weeks prior to minipump implantation (Fig. 1). Following this, animals were divided into matched treatment groups according to their anxiety phenotype and drinking behavior. Although there was no difference in anxiety-like behavior between groups at baseline (*F* = 2.673, *P* > 0.05) (Fig. 2A and B), following 4 weeks of drug treatment a significant overall effect of treatment condition on time spent in the open arms of the plus maze was observed (*F* = 7.138, *P* < 0.01). Post hoc analysis revealed that animals receiving prazosin (*n* = 6) spent significantly more time exploring the open arms than animals receiving propranolol (*n* = 7, *q* = 5.095, *P* < 0.01) or vehicle (*n* = 7, *q* = 4.483, *P* < 0.05) (Fig. 2D). Likewise, animals receiving duloxetine (*n* = 6) spent more time on the open arms that those in the propranolol (*q* = 4.713, *P* < 0.01) and vehicle (*q* = 4.101, *P* < 0.01) groups (Fig. 2D). Consistently, an analysis of the percent time spent in the open arms revealed a significant difference between group (*F* = 7.138, *P* < 0.01), and post hoc analysis revealed that animals receiving prazosin or duloxetine spent significantly more time exploring the open arms than animals receiving propranolol (*P* < 0.05) (data not shown). There was also an overall effect of treatment on open arm entries (*F* = 5.305, *P* < 0.01), with post hoc analysis revealing that prazosin-treated animals exhibited a higher percentage of open arm entries than either propranolol (*q* = 4.928, *P* < 0.01) or vehicle-treated animals (*q* = 3.937, *P* < 0.05) (Fig. 2E). Duloxetine-treated rats exhibited a higher percentage of open arm entries than those receiving propranolol (*q* = 3.738, *P* < 0.05); however, the difference in open-arm entries in duloxetine versus vehicle-related animals did not achieve significance (*q* = 2.747, *P* < 0.06) (Fig. 2E). In contrast, treatment condition did not alter the number of closed arm entries (*F* = 0.906, *P* > 0.05), a measure of nonspecific locomotor activity (Fig. 2F). In keeping with the above results, a two-way repeated measure ANOVA revealed no significant effect of drug treatment on general locomotor activity in the open field test (*F* = 0.641, *P* > 0.05) (Table S1). No differences in anxiety-like behavior (assessed as percent time spent in the margins versus the center of the open field) were observed on this assay, however; a two-way repeated measures ANOVA revealed a significant main effect of time (*F* = 2.621, *P* < 0.01) but no main effect of treatment (*F* = 1.918, *P* > 0.05) on time spent exploring the center of the novel open field environment, relative to the perimeter (Table S1).

### Prazosin and duloxetine decrease ethanol intake

All animals exhibited a progressive increase in ethanol intake and preference during the first week of intermittent access, as has been previously reported using this model (Simms 2008; Sinclair and Li, 1989; Wise, 1973). Prior to minipump implantation, animals were assigned to groups which were matched according to total intake on the days when ethanol was available; thus, there were no significant differences in baseline ethanol self-administration behavior. However, one to two animals in each group were eliminated from final analyses due to surgical or minipump-related complications. Likewise, one animal in each group was excluded from final analyses because these animals averaged < 1 g/kg daily ethanol intake throughout the 8-week baseline drinking period. However, this attrition had no significant effect on daily ethanol intake between groups (one-way ANOVA comparing 24 h g/kg intake at baseline, *F* = 0.31, *P* > 0.05).

**Figure 3:**
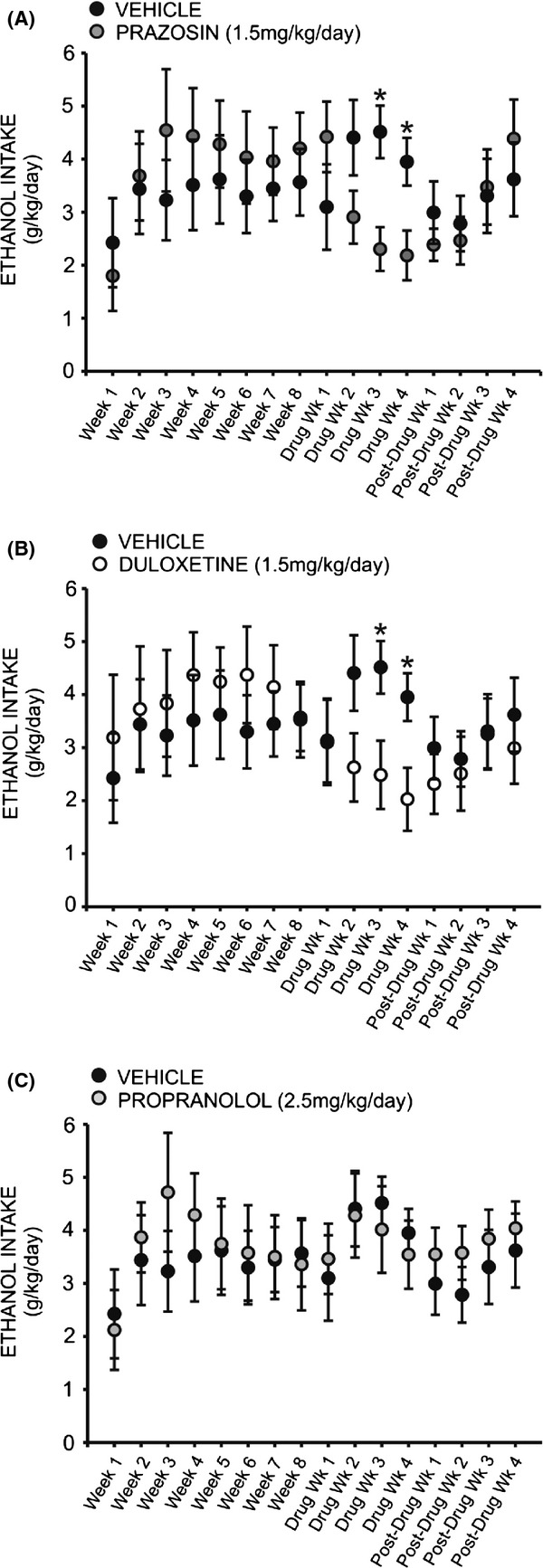
Chronic treatment with prazosin (A) or duloxetine (B) decreases intermittent ethanol (EtOH) self-administration relative to vehicle-treated conspecifics. Graphs represent 24 h (daily) EtOH intake each week for eight consecutive baseline weeks, followed by 4 week treatment with prazosin (*n* = 6, 1.5 mg/kg/day), duloxetine (*n* = 6, 1.5 mg/kg/day), propranolol (*n* = 7, 2.5 mg/kg/day), or vehicle (*n* = 7, 10% DMSO), and four additional posttreatment weeks. Animals had access to EtOH (20% v/v) 3 days a week for 24 h; weekly intake was averaged for each rat. Both prazosin (A) and duloxetine-treated (B) rats self-administered significantly less EtOH than vehicle-treated animals by the third week of drug delivery; this effect persisted through the final treatment week and was abolished following cessation of treatment (*, significant difference relative to vehicle-treated animals, two-way ANOVAs comparing treatment across time and Newman–Keuls post hoc tests, *P* < 0.05). Daily ethanol intake among propranolol-treated animals did not differ significantly from controls at any point (C) (two-way ANOVA comparing propranolol-treated animals to vehicle-treated conspecifics, *P* > 0.05).

A two-way repeated measures ANOVA comparing daily ethanol intake across all treatment groups revealed a significant interaction effect (*F* = 3.388, *P* < 0.05), and a follow-up two-way repeated measures ANOVA comparing prazosin and vehicle-treated animals at baseline and across 4 weeks of drug treatment revealed a significant treatment by time interaction (*F* = 3.094, *P* < 0.01) (Fig. 3A). Post hoc analysis revealed no difference between the two groups during the last week of baseline drinking (*q* = 1.042, *P* > 0.05), nor the first week of drug treatment (*q* = 2.159, *P* > 0.05). However, by the second week of drug treatment prazosin-treated animals were drinking marginally less than vehicle controls (*q* = 2.453, *P* < 0.09), and by week three they were drinking significantly less (*q* = 3.613, *P* < 0.05); this difference persisted through week four of drug treatment (*q* = 2.886, *P* < 0.05). Similarly, a two-way repeated measures ANOVA comparing daily ethanol intake in duloxetine and vehicle-treated animals revealed a significant interaction of treatment and time (*F* = 3.388, *P* < 0.05) (Fig. 3B). Post hoc analyses exposed no difference in drinking between groups at baseline (*q* = 0.057, *P* > 0.05) nor following 1 week of drug exposure (*q* = 0.0524, *P* > 0.05). However, by the second week of drug treatment duloxetine-treated animals were drinking marginally less ethanol than vehicle-treated conspecifics (*q* = 2.702, *P* < 0.07); this effect was significant following 3 weeks of drug exposure (*q* = 3.085, *P* < 0.05) and persisted through drug week 4 (*q* = 2.932, *P* < 0.05). There was no significant effect of propranolol treatment on ethanol intake, compared to vehicle (*F* = 1.437, *P* > 0.05) (Fig. 3C).

**Figure 4:**
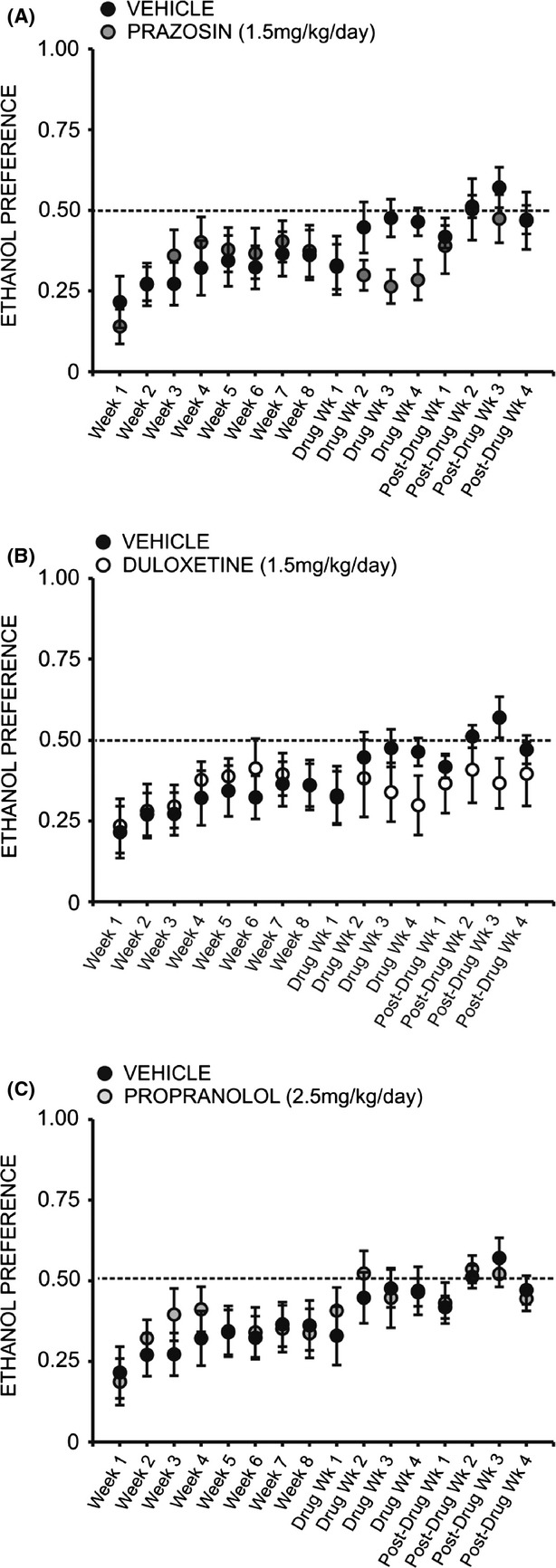
Chronic treatment with prazosin, duloxetine, or propranolol does not decrease preference for ethanol (EtOH) relative to vehicle-treated conspecifics. Graphs represent 24 h (daily) EtOH preference relative to water each week for eight consecutive baseline weeks, followed by 4 week treatment with prazosin (*n* = 6, 1.5 mg/kg/day), duloxetine (*n* = 6, 1.5 mg/kg/day), propranolol (*n* = 7, 2.5 mg/kg/day), or vehicle (*n* = 7, 10% DMSO), and four additional posttreatment weeks. Animals had access to EtOH (20% v/v) 3 days a week for 24 h; weekly EtOH preference was averaged for each rat. Treatment with prazosin (A), duloxetine (B), or propranolol (C) did not significantly decrease ethanol preference, relative to vehicle-treated conspecifics two-way ANOVAs comparing treatment across time, *P* > 0.05).

**Figure 5:**
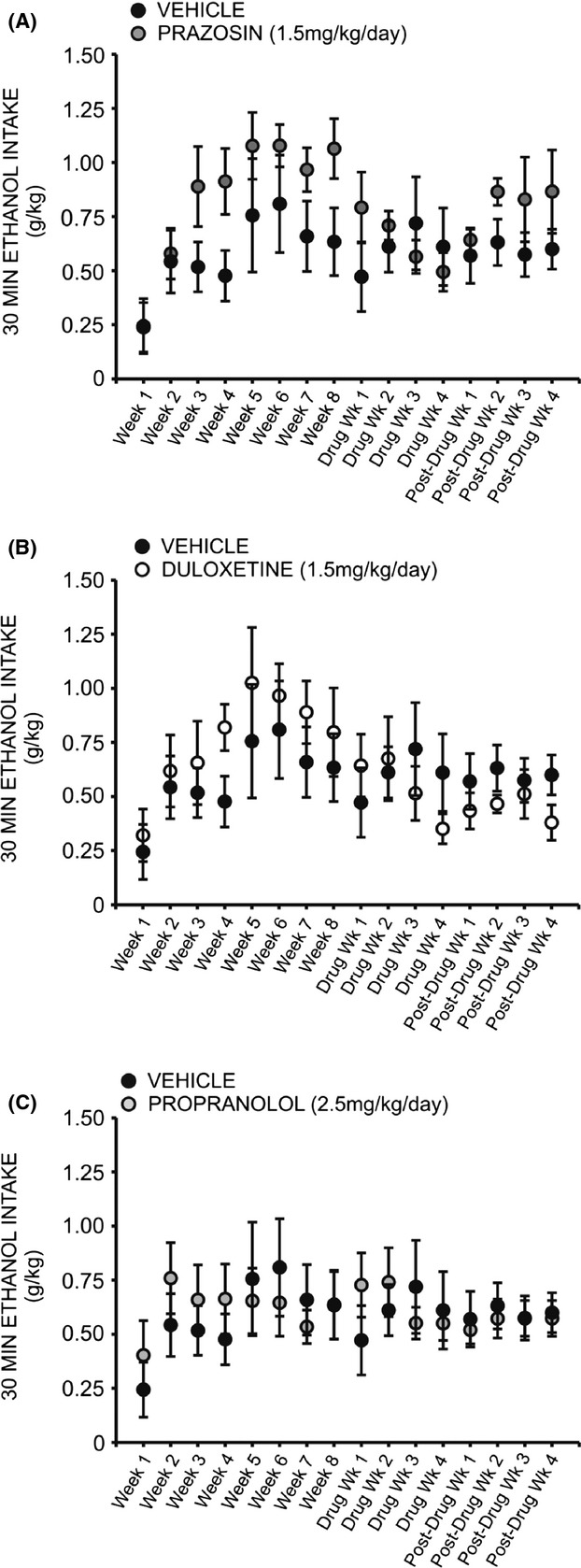
Chronic treatment with prazosin, duloxetine, or propranolol does not decrease binge-like ethanol (EtOH) self-administration relative to vehicle-treated conspecifics. Graphs represent EtOH intake in the first 30 min of daily access (binge-like intake) each week for eight consecutive baseline weeks, followed by 4 week treatment with prazosin (*n* = 6, 1.5 mg/kg/day), duloxetine (*n* = 6, 1.5 mg/kg/day), propranolol (*n* = 7, 2.5 mg/kg/day), or vehicle (*n* = 7, 10% DMSO), and four additional posttreatment weeks. Animals had access to EtOH (20% v/v) 3 days a week for 24 h; weekly binge-like intake was averaged for each rat. Treatment with prazosin (A), duloxetine (B), or propranolol (C) had no signiricant effect on binge-like ethanol intake relative to vehicle-treated animals, two-way ANOVAs comparing treatment across time, *P* > 0.05).

A two-way repeated measures ANOVA comparing daily ethanol preference across all treatment groups revealed a significant interaction effect (*F* = 1.905, *P* < 0.05). Interestingly, a two-way repeated measures ANOVA comparing preference for ethanol over water following treatment with prazosin or vehicle did not reveal a significant interaction of treatment and time (*F* = 2.378, *P* > 0.05) (Fig. 4A); likewise, no significant interaction was observed when comparing ethanol preference in duloxetine-treated animals (Fig. 4B) (*F* = 1.611, *P* > 0.05) and propranolol-treated animals (Fig. 4C) (*F* = 0.975, *P* > 0.05) to vehicle-treated conspecifics. A two-way repeated measures ANOVA comparing binge-like drinking in the first 30 min of daily access revealed a significant interaction of treatment and time (*F* = 1.960, *P* < 0.05), but there was also no significant effect of prazosin (Fig. 5A), duloxetine (Fig. 5B), or propranolol (Fig. 5C) on ethanol intake in the first 30 min of daily exposure, relative to vehicle treatment (prazosin *F* = 0.722, *P* > 0.05, duloxetine *F* = 0.507, *P* > 0.05, propranolol *F* = 0.076, *P* > 0.05).

**Figure 6:**
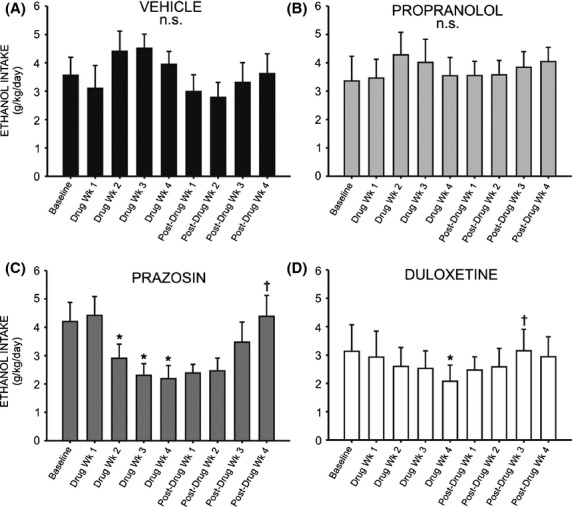
Chronic treatment with prazosin or duloxetine decreases intermittent ethanol (EtOH) self-administration relative to pretreatment baseline. Graphs represent 24 h (daily) EtOH intake during the last of eight consecutive baseline weeks, followed by 4 week treatment with prazosin (C) (*n* = 6, 1.5 mg/kg/day), duloxetine (D) (*n* = 6, 1.5 mg/kg/day), propranolol (B) (*n* = 7, 2.5 mg/kg/day), or vehicle (A) (*n* = 7, 10% DMSO), and four additional posttreatment weeks. Animals had access to EtOH (20% v/v) 3 days a week for 24 h; weekly intake was averaged for each rat. Treatment with prazosin resulted in reduced EtOH self-administration by the second week of drug delivery, and this difference persisted through the fourth treatment week (B). Duloxetine-treated animals consumed significantly less EtOH during the fourth week of drug delivery, while propranolol and vehicle had no significant effect on EtOH intake, relative to baseline (D). Following cessation of treatment, animals who had received prazosin (C) or duloxetine (D) returned to pretreatment levels of EtOH self-administration (*, significant difference relative to the last baseline week, one-way repeated measures ANOVAs and Newman–Keuls post hoc tests, *P* < 0.05; †, significant difference relative to the last treatment week, one-way repeated measures ANOVAs and Newman–Keuls post hoc tests, *P* < 0.05).

We also examined the within-group effect of each treatment on ethanol intake using one-way repeated measures ANOVAs and Neuman–Keuls post hoc tests. Analysis of 24 h intake at baseline versus each week of duloxetine treatment revealed that ethanol intake was significantly decreased across the treatment weeks (*F* = 3.124, *P* < 0.01), and post hoc analysis revealed that duloxetine-treated rats drank significantly less ethanol during the fourth week of drug delivery, relative to baseline (*q* = 5.417, *P* < 0.01) (Fig. 6D). Prazosin also decreased drinking relative to baseline (Fig. 6C) (*F* = 6.975, *P* < 0.001), and this difference was significant when comparing baseline to drug weeks 2 through 4 (*q* = 3.33, *P* < 0.05, *q* = 5.321, *P* < 0.01, and *q* = 5.656, *P* < 0.01, respectively). There was no significant effect of drug treatment following 4 weeks of treatment with propranolol (Fig. 6B) (*F* = 2.50, *P* > 0.05) or vehicle (Fig. 6A) (*F* = 1.724, *P* > 0.05).

We next investigated whether cessation of drug treatment resulted in significantly increased ethanol intake, comparing drinking at week 4 of drug treatment to weeks 1 through 4 of post-treatment intake in each group. Following cessation of duloxetine treatment, there was a return to baseline ethanol intake levels (*F* = 4.087, *P* < 0.05), and post hoc tests show that intake was significantly increased versus the final week of drug treatment by week 3 of posttreatment ethanol access (*q* = 4.977, *P* < 0.05) (Fig. 6D). Likewise, following cessation of prazosin delivery, 24 h ethanol intake increased (*F* = 7.864, *P* < 0.01), and post hoc tests revealed that by week 4 of posttreatment ethanol access animals were drinking significantly more than they had during the final week of drug treatment (*q* = 6.170, *P* < 0.05) (Fig. 6C).

**Figure 7:**
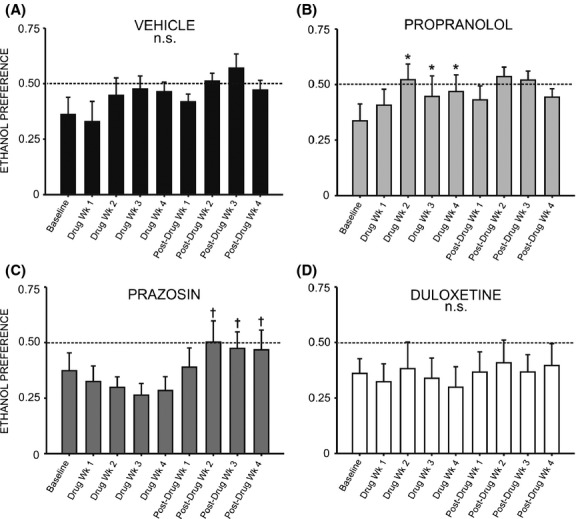
Chronic treatment with propranolol increases preference for ethanol (EtOH) relative to pretreatment baseline. Graphs represent 24 h (daily) EtOH preference during the last of eight consecutive baseline weeks, followed by 4 week treatment with prazosin (*n* = 6, 1.5 mg/kg/day), duloxetine (*n* = 6, 1.5 mg/kg/day), propranolol (*n* = 7, 2.5 mg/kg/day), or vehicle (*n* = 7, 10% DMSO), and four additional posttreatment weeks. Animals had access to EtOH (20% v/v) 3 days a week for 24 h; weekly intake was averaged for each rat. Treatment with propranolol (B) significantly increased EtOH preference by the second week of drug delivery; this effect persisted throughout treatment and was not reversed following removal of the drug. Animals in the duloxetine (D), prazosin (C), and vehicle-treated (A) groups did not exhibit significant alterations in EtOH preference during treatment, although following cessation of prazosin delivery (C), preference for EtOH increased significantly relative to the last week of treatment and remained elevated for the duration of the study (*, significant difference relative to the last baseline week, one-way repeated measures ANOVAs and Newman–Keuls post hoc tests, *P* < 0.05; †, significant difference relative to the last treatment week, one-way repeated measures ANOVAs and Newman–Keuls post hoc tests, *P* < 0.05).

We did not observe any change in preference for ethanol over water over the course of drug delivery when comparing baseline preference to each week of duloxetine treatment (Fig. 7D) (*F* = 0.894, *P* > 0.05) or prazosin treatment (Fig. 7C) (*F* = 0.739, *P* > 0.05); however, following cessation of drug delivery animals previously receiving prazosin exhibited a significant increase in ethanol preference relative to the final week of drug treatment (*F* = 4.887, *P* < 0.05) (Fig. 7C). Post hoc analysis revealed that ethanol preference was significantly increased 2 weeks following minipump removal (*q* = 4.737, *P* < 0.05) and remained elevated through weeks 3 and 4 (*q* = 4.747, *P* < 0.05 and *q* = 5.459, *P* < 0.05, respectively). Interestingly, propranolol treatment increased ethanol preference over the course of drug delivery (Fig. 7B) (*F* = 8.295, *P* < 0.001). Post hoc analysis revealed that by treatment week 2, preference for ethanol was increased relative to baseline (*q* = 7.687, *P* < 0.001), and this increase persisted through the third (*q* = 4.555, *P* < 0.05) and fourth (*q* = 5.479, *P* < 0.01) weeks of propranolol treatment (Fig. 7B).

**Figure 8:**
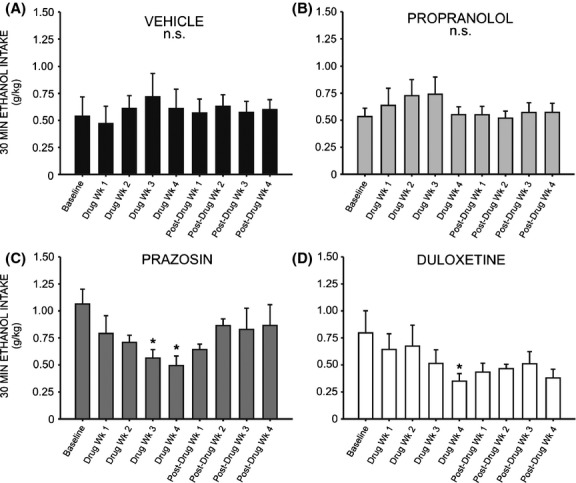
Chronic treatment with prazosin or duloxetine decreases binge-like ethanol (EtOH) self-administration relative to pretreatment baseline. Graphs represent EtOH intake in the first 30 min of daily access (binge-like intake) during the last of eight consecutive baseline weeks, followed by 4 week treatment with prazosin (*n* = 6, 1.5 mg/kg/day), duloxetine (*n* = 6, 1.5 mg/kg/day), propranolol (*n* = 7, 2.5 mg/kg/day), or vehicle (*n* = 7, 10% DMSO), and four additional posttreatment weeks. Animals had access to EtOH (20% v/v) 3 days a week for 24 h; weekly intake was averaged for each rat. Binge-like EtOH intake was significantly reduced by the third week of prazosin delivery (C), and by the final week of treatment with duloxetine (D). Propranolol (B) and vehicle-treated (A) animals did not change their binge-like EtOH intake in response to treatment (*, significant difference relative to the last baseline week, one-way repeated measures ANOVAs and Newman–Keuls post hoc tests, *P* < 0.05; †, significant difference relative to the last treatment week, one-way repeated measures ANOVAs and Newman–Keuls post hoc tests, *P* < 0.05).

Correspondingly, one-way repeated measures ANOVAs revealed a significant change in binge-like intake (g/kg in the first 30 min of daily ethanol access) relative to baseline drinking following both prazosin (Fig. 8D) and duloxetine treatment (Fig. 8D) (*F* = 3.379, *P* < 0.05 and *F* = 3.394, *P* < 0.05, respectively). Post hoc analyses revealed no significant change in 30 min intake during the first 2 weeks of prazosin treatment (*q* = 2.244, *P* > 0.05 week one, and *q* = 2.929, *P* > 0.05 week two); however, by week 3 binge-like drinking had significantly decreased as compared to baseline (*q* = 4.119, *P* < 0.05) and this persisted through treatment week 4 (*q* = 4.700, *P* < 0.05). Similarly, binge-like intake was not significantly decreased during the first 3 weeks of duloxetine treatment relative to baseline (*q* = 1.669, *P* > 0.05 week one; *q* = 1.323, *P* > 0.05 week two; *q* = 3.060, *P* > 0.05 week three) (Fig. 8). During week 4, however, drinking during the first 30 min of daily access decreased significantly (*q* = 4.835, *P* < 0.05). Neither propranolol (Fig. 8B) nor vehicle treatment (Fig. 8A) decreased binge-like intake relative to baseline at any time point (*F* = 2.009, *P* > 0.05 and *F* = 0.787, *P* > 0.05, respectively). It is important to note that while we did not measure blood ethanol concentrations, we have reported previously that blood ethanol levels measured following the first thirty minutes of daily ethanol access are strongly correlated with ethanol intake during this period (Chappell et al. 2013).

**Figure 9:**
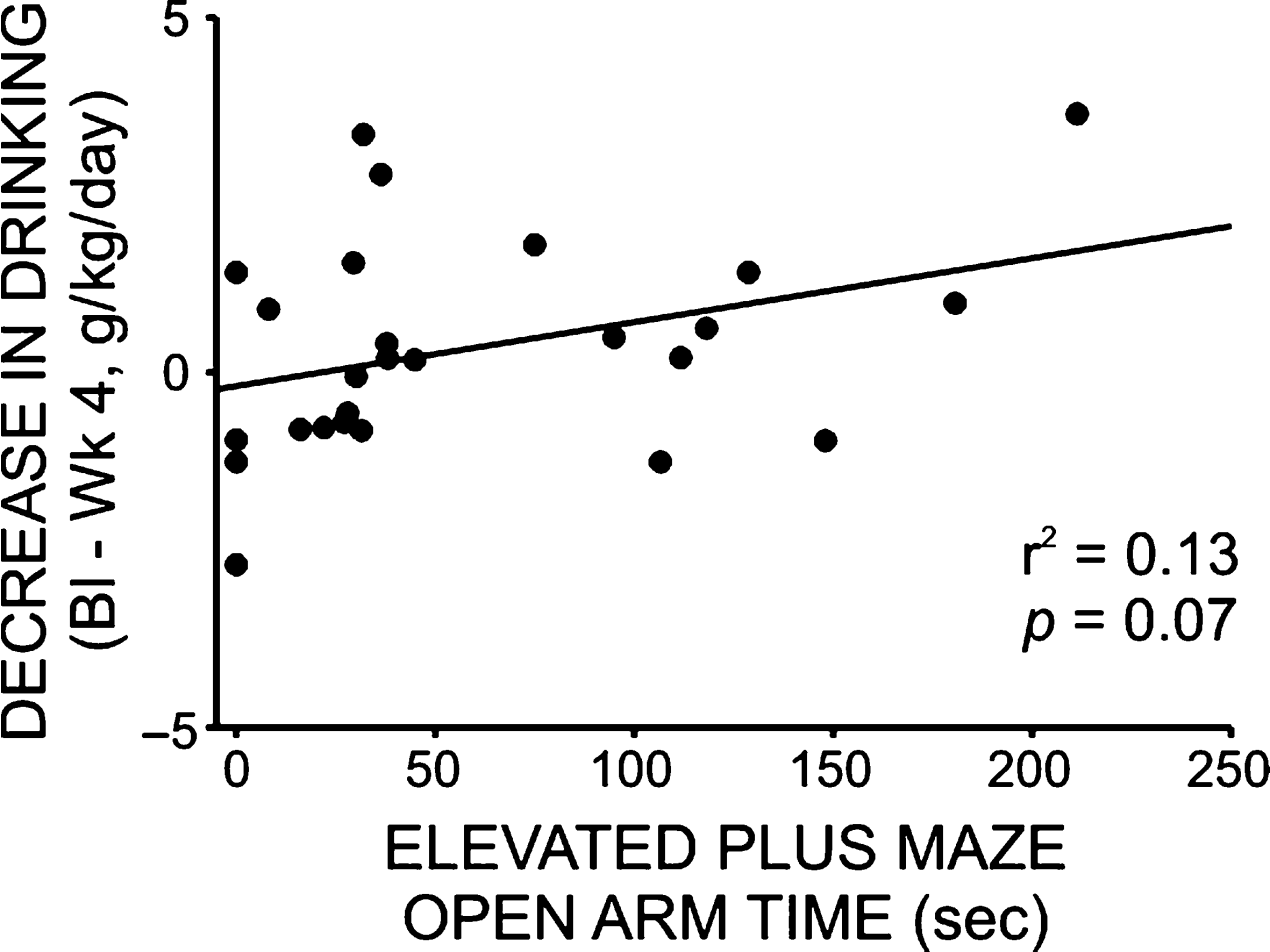
Change in daily ethanol administration (baseline – treatment week 4) was not correlated with posttreatment anxiety-like behavior on the elevated plus maze (open arm time), although there was a modest trend toward a positive relationship between these variables. Animals from all treatment groups (propranolol, 2.5 mg/kg/day; prazosin 1.5 mg/kg/day; duloxetine 1.5 mg/kg/day; vehicle 10% DMSO in sterile saline) had access to EtOH (20% v/v) 3 days a week for 24 h; weekly intake was averaged for each rat. At the end of a 4 week drug delivery period, all animals were tested for anxiety-like behavior on the elevated plus maze. The change in each animal's daily intake following 4 weeks of drug delivery (24 h g/kg baseline – 24 h g/kg treatment week 4) is plotted on the y-axis, and time spent in the open arms of the elevated plus maze is plotted on the x-axis. Although the relationship between these factors was not statistically significant, a positive trend emerged (*r*^2^ = 0.13, *F* = 3.441, *P* = 0.07).

Finally, to assess directly the relationship between ethanol intake and anxiety-like behavior, we ran a Pearson correlation comparing time spent on the open arms of the elevated plus maze following drug delivery to the change in daily ethanol intake following 4 weeks of treatment with prazosin, duloxetine, propranolol, or vehicle (baseline g/kg EtOH— treatment week 4 g/kg EtOH). Although the relationship between these factors was not statistically significant, a positive trend was noted (*r*^2^ = 0.13, *F* = 3.441, *P* = 0.07) (Fig. 9).

A two-way repeated measures ANOVA comparing water intake at baseline to each week of drug treatment revealed a significant interaction of treatment and time (*F* = 2.367, *P* < 0.05). A follow-up two-way repeated measures ANOVA comparing duloxetine and vehicle treated animals was non-significant (*F* = 0.291, *P* > 0.05) (Fig. S1). A comparison of prazosin treated animals and vehicle treated conspecifics was likewise non-significant (*F* = 1.716, *P* > 0.05) (Fig. S1). However, a repeated measures ANOVA comparing vehicle and propranolol treated animals did reveal a significant interaction (*F* = 3.167, *P* > 0.05), and post hoc analysis revealed that propranolol treated animals drank significantly less water during the first week of drug delivery, relative to baseline (*q* = 4.995, *P* < 0.05) (Fig. S1). Animals were weighted each day that ethanol was available, immediately prior to daily access. A two-way repeated measures ANOVA comparing average weight across groups at baseline to each week of drug treatment revealed a significant effect of time (*F* = 36.781, *P* < 0.001) but no main effect of treatment (*F* = 0.207, *P* > 0.05) or interaction effect (*F* = 0.999, *P* > 0.05) (Fig. S2).

## Discussion

The results of these studies confirm and expand upon previous reports that pharmacological treatments believed to decrease central noradrenergic signaling may be effective in diminishing ethanol self-administration and attenuating anxiety-like behavior. Chronic administration of the *α*-1 adrenoreceptor antagonist, prazosin, decreased ethanol intake relative to vehicle by the third week of treatment, and this difference persisted through the final week of drug administration. Furthermore, within-subjects analysis revealed that 1 week of prazosin delivery significantly and persistently decreased ethanol intake relative to baseline. The SNRI duloxetine likewise decreased ethanol intake throughout the third and fourth weeks of drug delivery, relative to time-matched controls receiving vehicle. Similarly, when compared to baseline, duloxetine-treated animals exhibited significantly suppressed drinking by the fourth week of treatment. Furthermore, by week 3 of prazosin treatment subjects' binge-like ethanol intake in the first 30 min of daily access had significantly decreased relative to baseline; 30 min intake was not significantly affected relative to vehicle-treated conspecifics, however. Likewise, duloxetine decreased binge-like drinking relative to baseline by the final week of treatment, although this measure of intake was not significantly decreased relative to vehicle-treated animals at any time point. Following cessation of drug delivery, both groups reverted back to pretreatment ethanol intake. Neither the *β*1/2 adrenoreceptor antagonist propranolol nor vehicle significantly decreased ethanol intake at any point, and no treatment decreased preference for ethanol over water. Importantly, we report that anxiety-like behavior on the elevated plus maze was decreased following 4-week treatment with prazosin or duloxetine, relative to vehicle-treated animals. Finally, plus maze activity after 4 weeks of treatment was positively correlated with decreased ethanol intake following 4 weeks of drug delivery; this trend, although not significant, is consistent with our hypothesis that disrupted noradrenergic signaling increases anxiety and resultant ethanol intake.

To our knowledge, this is the first study to directly seek an association between the ability of chronically administered pharmacotherapeutics to reduce anxiety-like behaviors and ethanol self-administration in the same animals. Our results confirm that long-term treatment with drugs thought to decrease noradrenergic signaling reduces both ethanol intake and the expression of anxiety-like behavior in well-validated rodent models. The observed effectiveness of prazosin and duloxetine in reducing drinking while apparently inducing anxiolysis is very encouraging, as these findings correspond with their efficacy in attenuating these behaviors among clinical populations (Liappas et al. 2005; Yoon et al. 2006; Simpson et al. 2009; Fox et al. 2012; Petrakis et al. 2012). Our results are consistent with previous reports that acute administration of noradrenergic agents reduces drinking-related behaviors in animals models of ethanol intake (Saglam et al. 2004; Ji et al. 2008; Walker et al. 2008; Rasmussen et al. 2009; Gilpin and Koob 2010; Simon O'Brien et al. 2011; Verplaetse et al. 2012). We likewise corroborate recent reports that chronically administered prazosin decreases ethanol intake in rodent models of ethanol self-administration (Froehlich et al. 2013a,b), and extend these findings by confirming that prazosin treatment concurrently decreases anxiety.

It is interesting to note that while these pharmacological interventions decreased anxiety-like behavior on the elevated plus maze, they had no discernible effect on anxiety measures assessed by the open field test; however, perhaps this result is not surprising, as the results of a literature review suggest that this assay may not be suitable for measuring the effectiveness of all anxiolytic compounds (Prut and Belzung 2003). Regardless, to our knowledge, these studies are the first to confirm that reduced drinking resulting from chronic treatment with drugs that putatively decrease central noradrenergic signaling is accompanied by significantly diminished anxiety-like behavior. Regarding our use of the elevated plus maze, we acknowledge that there is some debate as to whether retesting on this assay is appropriate. Conventionally, it has been argued that while behavior expressed on first exposure to the assay assesses anxiety-like behavior, performance on a subsequent test reflects learned aversion and is not sensitive to anxiolytic drugs (File et al., 1990). However, recent studies have provided strong evidence that this behavioral shift is abolished if the two exposures to the plus maze are sufficiently separated in time. For example, Schneider and colleagues (Schneider et al. 2011) demonstrated that 28 days between tests was sufficient to produce reliable re-assessment of anxiety-like behaviors on the elevated plus maze. Specifically, these authors observed a significant positive correlation between trials for time on the open arms and open arm entries, suggesting that this is indeed a measure of trait anxiety-like behavior. Moreover, the “one-trial” tolerance that has frequently been reported with this assay may be restricted to benzodiazepines, as we and others have found no evidence of this phenomenon in Long Evans rats with other anxiolytic medications (McCool and Chappell 2007; Silberman et al. 2010).

Interestingly, prior research in our lab has revealed that adult male Long-Evans rats acquired from a commercial supplier display a relatively anxiogenic phenotype (Chappell et al. 2013). These animals self-administer ethanol at levels comparable with animals that have been chronicall stressed throughout adolescence, and likewise exhibit equivalent anxiety-like behavior on the open plus maze (versus age-matched animals that were not subjected to the developmental stressor). Additionally, evidence from our lab suggests that prazosin, propranolol, and duloxetine, when administered acutely, are more effective at decreasing drinking in stressed animals than nonstressed controls (Skelly et al. 2012), and other groups have reported that prazosin is more effective at decreasing drinking in ethanol-dependent animals than nondependent subjects (Walker et al. 2008). Likewise, the SNRI milnacipran reportedly reduces self-administration in ethanol-dependent rats at doses that are ineffective in nondependent rats (Simon O'Brien et al. 2011). These findings are consistent with the hypothesis that chronic stress and/or prolonged ethanol intake can dramatically alter central noradrenergic signaling, and these neurobiological alterations may likewise render the stress system more sensitive to pharmacological treatments that decrease NE signaling. As such, it will be important to examine whether the compounds tested in this study are less efficacious when administered to animals expressing “normal” anxiety-like behavior and ethanol intake, toward the goal of more directly assessing whether the effects observed herein are directly related to stress-induced disregulation of noradrenergic signaling.

Although we did not directly assess the neurobiological mechanisms by which these treatments reduced drinking and anxiety, we can speculate as to how they are affecting these behaviors. Previous studies have shown that blocking central noradrenergic signaling reduces ethanol self-administration in rats (Amit et al. 1977; Davis et al. 1978; Mason et al. 1979; Corcoran et al. 1983). Norepinephrine acts presynaptically at α-2 receptors in the LC; thus, duloxetine may reduce NE signaling by decreasing output from this region. In support of this hypothesis, in vivo recordings have revealed that chronic SNRI treatment leads to decreased LC firing in rats (West et al. 2009), and pharmacological activation of α-2 adrenoceptors has been shown to reduce both ethanol self-administration and stress-induced reinstatement of ethanol seeking in rats (Le et al. 2005). Likewise, NE exerts its excitatory effects at postsynaptic *α*-1 and *β*-1/2 adrenoreceptors in many brain regions, including those involved in stress and anxiety. Relevant to these studies, increased NE release in the bed nucleus of the stria terminalis (BNST) has been observed during withdrawal from drugs of abuse (Delfs et al. 2000; Aston-Jones and Harris 2004). The resulting increased activation of postsynaptic *β*-1/2 and *α*-1 adrenoreceptors might, in turn, increase BNST output and result in increased anxiety; as such, prazosin may decrease anxiety-related ethanol intake by diminishing the excitatory effects of NE in this region (Walker et al. 2008).

It is interesting to note that neither prazosin nor duloxetine was immediately effective in decreasing drinking. These findings are largely consistent with previous studies; Froehlich et al. (2013b) report that the dose of prazosin used herein, when administered daily, did not significantly reduce drinking until the fourth treatment week. Likewise, duloxetine did not significantly decrease ethanol intake relative to baseline until the fourth week of drug delivery, which is consistent with the delayed time-course of antidepressant effectiveness in humans. As discussed above, we have shown that these doses are sufficient to decrease ethanol intake when given in an acute, bolus injection; that they differently affect behavior when administered chronically further strengthens the rationale for studying the effects of these compounds across many weeks of continual administration, as opposed to analyzing acute effects which bear little relationship with clinical treatment conditions. Ongoing studies in our lab and others have demonstrated that rats raised in groups during adolescence exhibit lower levels of anxiety-like behavior in adulthood and self-administer less ethanol than socially isolated or commercially sourced adult rats, such as the animals used for these studies (Chappell et al. 2013; Yorgason et al. 2013). On the basis of the findings reported herein, we plan to not only compare the effectiveness of chronic prazosin and duloxetine in group housed and socially isolated rats, but also to examine the neuroadaptive changes resulting from prolonged treatment with these pharmacotherapeutics, toward the goal of elucidating how noradrenergic signaling is disrupted by stress-induced cyclic intoxication and withdrawal and righted by prolonged exposure to drugs which decrease NE's excitatory effects.

Interestingly, chronic propranolol treatment did not appear to decrease drinking nor alter anxiety measures. This is surprising, as propranolol has been shown to acutely decrease drinking in rats (Gilpin and Koob 2010; Skelly et al. 2012), and has been used clinically to treat anxiety associated with alcohol withdrawal (Bailly et al. 1992). Propranolol also controls some of the clinical signs of withdrawal (Carlsson and Johansson 1971; Zilm et al. 1975), and significantly decreases circulating noradrenaline levels in humans during ethanol withdrawal (Sellers et al. 1976). Furthermore, there is evidence of increased *β*-adrenergic receptor sensitivity in the alcohol withdrawal state in rats (Banerjee et al. 1978), and *β*2-adrenoreceptor expression is reportedly upregulated in rats with a history of ethanol self-administration (Rimondini et al. 2002). However, chronic ethanol exposure has also been reported to uncouple the *β*-2 adrenergic receptor from its G-protein in the frontal cortex of both rats and humans (Gurguis et al. 1998, 1999). Interestingly, while the sensitivity of *β*-adrenergic receptors increases in proportion to anxiety levels in a normal population (Kang and Yu 2005), *β*-adrenergic receptor sensitivity is lower among individuals under chronic stress (Dimsdale et al. 1994) or suffering from clinical anxiety disorders (Brown et al. 1988). Thus, chronic ethanol may be so disruptive to *β*-adrenergic signaling that pharmacotherapeutic agents acting on this system are rendered inert in the long-term. However, it is also possible that we simply did not administer a high enough dose of propranolol to see an effect of this drug chronically.

It is also important to note that while prazosin and duloxetine significantly decreased ethanol self-administration, neither drug affected ethanol preference at any point. These findings are not consistent with the decrease in both 24 h and binge-like ethanol intake observed in response to these treatments. One explanation for these seemingly inconsistent results might be that the doses of duloxetine and prazosin administered here were relatively low (1.5 mg/kg/day). We chose these doses based on their acute effects on ethanol self-administration when administered i.p.; however, while acute injections result in a bolus dose of drug delivered to the brain, these experiments allowed for much more protracted administration which may have attenuated their effectiveness. Future studies will examine higher doses of these compounds, and may reveal that increasing the amount of drug available results in decreased preference for ethanol over water.

In conclusion, our results indicate that chronic treatment with a low dose of prazosin or duloxetine significantly decreases ethanol self-administration, and that this decrease in drinking is accompanied by marked reductions in the expression of anxiety-like behavior. It is important to note that only the medications which reduced anxiety-like behavior also influenced ethanol intake. These findings suggest that chronic treatment with putative inhibitors of central noradrenergic signaling may assuage anxiety, thereby attenuating ethanol intake. These results have important implications for the treatment of comorbid anxiety and ethanol use disorders with known and available pharmacotherapeutics, as the ability to model potential treatments of anxiety and ethanol abuse disorders with well-tolerated drugs that are already on the market may allow researchers to identify the neural mechanisms underlying the effectiveness of these drugs in treating the symptoms of comorbid anxiety and alcohol use disorders. Understanding these mechanisms may, in turn, provide insight into the disrupted neural circuits underlying both disorders, toward the goal of developing more effective behavioral and pharmacological interventions.

## Supporting Information

**Figure S1.** Chronic treatment with propranolol decreases water intake relative to vehicle-treated conspecifics during the first week of treatment. **Figure S2.** Chronic treatment with prazosin, propranolol, or duloxetine has no effect on weight gain. **Table S1.** Chronic treatment with prazosin, propranolol, or duloxetine has no effect on anxiety-like behavior (center time, *P* > 0.05) or general locomotor activity (total distance traveled, *P* > 0.05) in the open field test.
