# Supplementary material for: Chronic treatment with prazosin or duloxetine lessens concurrent anxiety-like behavior and alcohol intake: evidence of disrupted noradrenergic signaling in anxiety-related alcohol use
Source: Brain Behav. 2014 Apr 14;4(4):468–83. doi: 10.1002/brb3.230 (PMC4128029; doi:10.1002/brb3.230)
Supplement: Supplementary file 1 [file brb30004-0468-SD1.docx]

Supplemental Information

Water Intake

A two-way repeated measures ANOVA comparing water intake at baseline to each week of drug treatment revealed a significant interaction of treatment and time (F = 2.367, *p* < 0.05). A follow-up two-way repeated measures ANOVA comparing duloxetine and vehicle treated animals was non-significant (ME treatment, F = 0.006, *p* > 0.05; ME time, F = 0.940, *p* > 0.05; Interaction, F = 0.291, *p* > 0.05) (Supp Fig 1). A comparison of prazosin treated animals and vehicle treated conspecifics was likewise non-significant (ME treatment, F = 0.703, *p* > 0.05; ME time, F = 1.675, *p* > 0.05; Interaction, F = 1.716, *p* > 0.05) (Supp Fig 1). However, a repeated measures ANOVA comparing vehicle and propranolol treated animals did reveal a significant interaction (F = 3.167, *p* > 0.05), and post hoc analysis revealed that propranolol treated animals drank significantly less water during the first week of drug delivery, relative to baseline (q = 4.995, *p* < 0.05) (Supp Fig 1).

Weight Gain

Animals were weighted each day that ethanol was available, immediately prior to daily access. A two-way repeated measures ANOVA comparing average weight across groups at baseline to each week of drug treatment revealed a significant effect of time (F = 36.781, *p* < 0.001) but no main effect of treatment (F = 0.207, *p* < 0.891) or interaction effect (F = 0.999, *p* < 0.457) (Supp Fig 2).

Figure Legends

**Supplemental Figure 1.** Chronic treatment with propranolol decreases water intake relative to vehicle-treated conspecifics during the first week of treatment. Graphs represent 24 hour (daily) water intake each week for eight consecutive baseline weeks, followed by four week treatment with prazosin (n = 6, 1.5 mg/kg/day), duloxetine (n = 6, 1.5 mg/kg/day), propranolol (n = 7, 2.5 mg/kg/day), or vehicle (n = 7, 10% DMSO), and four additional post-treatment weeks. Animals had access to EtOH (20% v/v) three days a week for twenty-four hours; weekly intake was averaged for each rat. Neither prazosin nor duloxetine treated rats self-administered significantly more water than vehicle treated animals at any time point (two-way repeated measures ANOVA comparing propranolol treated animals to vehicle treated conspecifics, p > 0.05). Daily water intake among propranolol treated animals was significantly increased during the first week of drug deliver, relative to vehicle treated animals, although this effect was abolished by the second treatment week (*, significant difference relative to vehicle treated animals, two-way ANOVAs comparing treatment across time and Newman-Keuls post hoc tests, *p* < 0.05). Daily ethanol intake among propranolol treated animals did not differ significantly from controls at any point

**Supplemental Figure 2.**  Chronic treatment with prazosin, propranolol, or duloxetine has no effect on weight gain. Graph represents average weight each week during baseline ethanol access, drug treatment, and post-treatment drinking. No treatment significantly altered weight gain (two-way repeated measures ANOVA comparing weight gain across time and treatment, *p* > 0.05).

**Supplemental Table 1.** Chronic treatment with prazosin, propranolol, or duloxetine has no effect on anxiety-like behavior (center time, *p* > 0.05) or general locomotor activity (total distance traveled, *p* > 0.05).

**
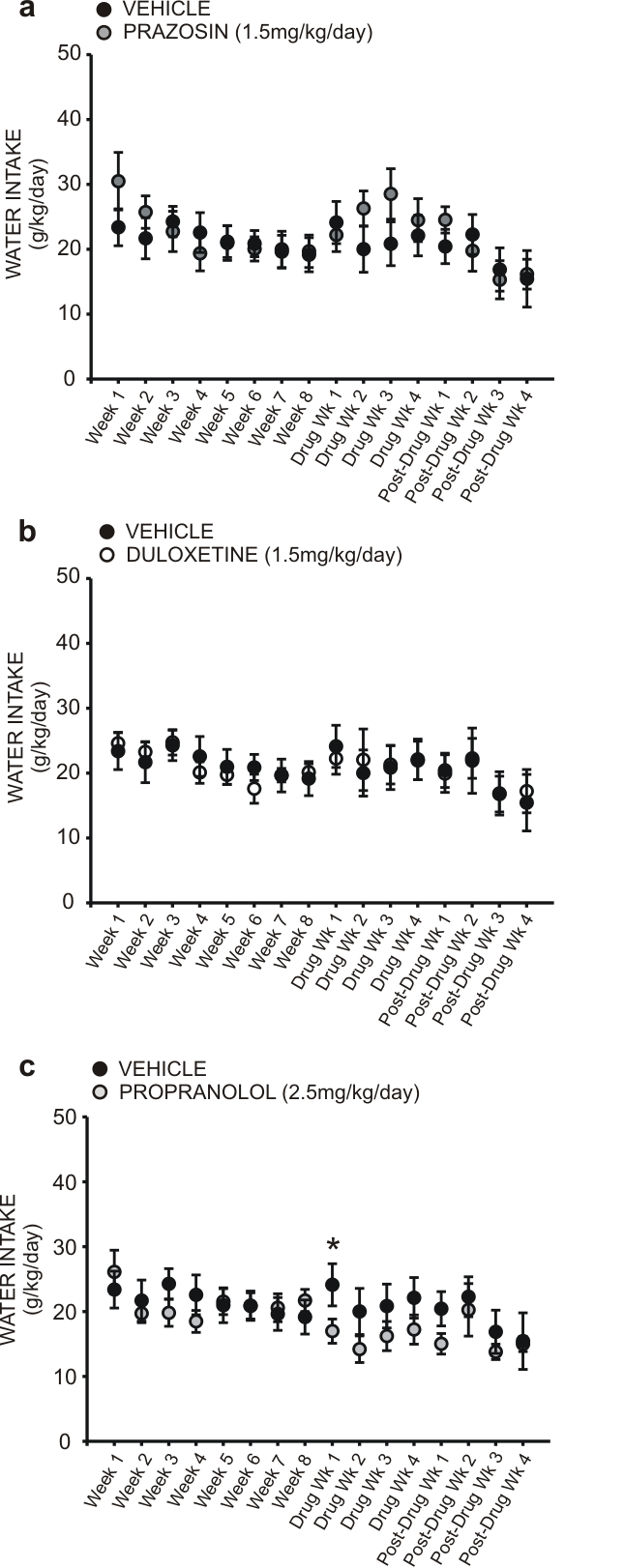
Supplemental Figure 1**

**
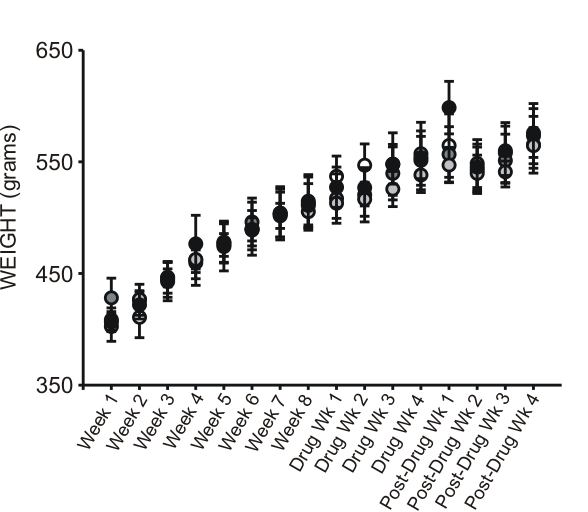
Supplemental Figure 2**

**Supplemental Table 1**

**
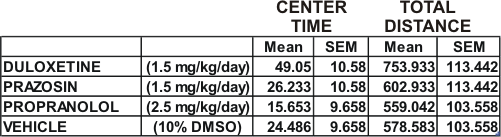
**
